# An empirical analysis of training protocols for probabilistic gene finders

**DOI:** 10.1186/1471-2105-5-206

**Published:** 2004-12-21

**Authors:** William H Majoros, Steven L Salzberg

**Affiliations:** 1 The Institute for Genomic Research, 9712 Medical Center Drive, Rockville, MD 20850, USA

## Abstract

**Background:**

Generalized hidden Markov models (GHMMs) appear to be approaching acceptance as a *de facto *standard for state-of-the-art *ab initio *gene finding, as evidenced by the recent proliferation of GHMM implementations. While prevailing methods for modeling and parsing genes using GHMMs have been described in the literature, little attention has been paid as of yet to their proper training. The few hints available in the literature together with anecdotal observations suggest that most practitioners perform maximum likelihood parameter estimation only at the local submodel level, and then attend to the optimization of global parameter structure using some form of *ad hoc *manual tuning of individual parameters.

**Results:**

We decided to investigate the utility of applying a more systematic optimization approach to the tuning of global parameter structure by implementing a global discriminative training procedure for our GHMM-based gene finder. Our results show that significant improvement in prediction accuracy can be achieved by this method.

**Conclusions:**

We conclude that training of GHMM-based gene finders is best performed using some form of discriminative training rather than simple maximum likelihood estimation at the submodel level, and that generalized gradient ascent methods are suitable for this task. We also conclude that partitioning of training data for the twin purposes of maximum likelihood initialization and gradient ascent optimization appears to be unnecessary, but that strict segregation of test data must be enforced during final gene finder evaluation to avoid artificially inflated accuracy measurements.

## Background

The number of generalized hidden Markov model (GHMM) gene finders reported in the literature has increased fairly dramatically of late [1-8], and the community is now contemplating various ways to extend this attractive framework in order to incorporate homology information, with a handful of such systems having already been built (e.g., [9-12]). GHMMs offer a number of clear advantages which would seem to explain this growth in popularity. Chief among these is the fact that the GHMM framework, being (in theory) purely probabilistic, allows for principled approaches to constructing, utilizing, and extending models for accurate prediction of gene structures.

While the decoding problem for GHMM gene finders is arguably well understood, being a relatively straightforward extension of the same problem for traditional HMMs and amenable to a Viterbi-like solution (albeit a more complex one), methods for optimally training a GHMM gene finder have received scant attention in the gene-finding literature to date. What information is available (e.g., [2,4]) seems to indicate that the common practice is to optimize the submodels of the GHMM independently, without regard for the optimality of the composite model.

The training of HMMs and GHMMs has traditionally been carried out using some form of *maximum likelihood estimation *(MLE). Baum-Welch training [13], which is an instance of the well-known *expectation maximization *(EM) procedure, is itself a form of MLE [14]. In the case of GHMM gene finders, one typically applies some form of MLE to each of the submodels (states) in the GHMM so as to render training features of each type (e.g., exon, intron, donor site) maximally likely under the induced (sub)model; i.e., maximizing:



for state *q *and for *S*_*i *_a feature of length *d*_*i *_from the state-*q*-specific training set *T*. The submodels are then merged into a composite model (i.e., the full GHMM) by observing transition probabilities between features in the training data corresponding to each of the GHMM states.

For example, an exon state in a GHMM can be trained by collecting *n*-gram statistics (i.e., counts of *n*-letter substrings) from known exon sequences and normalizing these into transition probabilities for an (*n*-1)^th^-order Markov chain [15]. Similarly, intron, intergenic, and untranslated region (UTR) states can be modeled by collecting appropriate statistics from corresponding sample features and using these to train individual content-scoring models, such as Markov chains, neural networks, decision trees, etc. Signal sensors for donor and acceptor splice sites and start and stop codons can be trained by aligning known signals of the appropriate type and counting nucleotide frequencies at each position within a fixed window around the signal; converting these counts to relative frequencies produces probability estimates for use in a weight matrix or similar type of model. Transition and duration probabilities can likewise be estimated by observing appropriate frequencies in training data. All of these estimation activities can be performed independently, resulting in a GHMM consisting of distinct subsets of maximum likelihood parameters.

Such an approach does not, however, attend to the global optimality of the GHMM as a whole. Ideally, one would like to maximize the expected accuracy of the gene finder on unseen data. A reasonable approximation to this ideal would be to maximize the average probability of the gene parses in the training set:



where the collection of model parameters making up the GHMM is denoted θ and the elements (*S*, φ) of the training set *T *comprise pairs of sequences *S *and their known parses φ. This argmax gives us the parameterization  under which the full gene *parses *(rather than the *sequences*) in the training set will be maximally likely (on average). Decomposing each parse φ into a series of (*q*_*i*_, *d*_*i*_) pairs, for state *q*_*i *_and state duration (i.e., feature length) *d*_*i*_, we get:



where *P*_*e*_, *P*_*t*_, and *P*_*d *_represent the emission, transition, and duration probabilities of the GHMM, respectively. Whereas the common MLE training procedure for GHMMs as described above optimizes the individual terms in the numerator of Equation 3 independently, the argmax above calls instead for these terms to be jointly tuned so as to optimize the entire ratio in parentheses. Intuitively, one can think of this alternate formulation as attempting to account for the process in the Viterbi algorithm (during later decoding) whereby the individual submodels "compete" for nucleotides (in the sense that each nucleotide can be emitted by only one submodel in any given parse, and the Viterbi algorithm chooses the final, predicted parse based on the values of the model parameters). Our hope is that by addressing the issue of submodel competition explicitly during parameter estimation, we will thereby empower the gene finder to better discriminate at a global sequence level between the features modeled by individual submodels in the GHMM, thereby producing more accurate gene predictions.

A similar optimization problem occurs in the field of speech recognition, in which systems of interacting acoustic models and language models are employed to optimally parse an audio stream into a series of discrete words. Interestingly, the trend in that field, starting with Bahl *et al*. in 1986 [16], has increasingly been away from the sole use of MLE and toward an alternative approach very similar to that prescribed by Equation 2 known as *global discriminative training *[17-19] or *conditional maximum likelihood *[20]. The problem also appears in a slightly different form in the related field of statistical natural language parsing, in which it has been suggested that global methods for optimizing competing stochastic grammar models may improve the accuracy of systems at the level of whole-sentence parses [21]. *Maximum discrimination HMMs *have already been applied successfully to problems in the realm of biological sequence analysis [22], though their use in gene finding has apparently not yet seen widespread adoption. To our knowledge, the only gene finder reported to use discriminative training is HMMgene [23], a gene finder based on a non-generalized HMM.

In light of these considerations, it is worth contemplating the possible gains in gene finder accuracy that might be obtained through the use of some form of discriminative training applied to a GHMM – that is, training aimed more directly at optimizing the ability of the gene finder to discriminate between exons and non-exons, thereby improving the expected accuracy of the gene finder's predictions. Anecdotal evidence already suggests that investigation of such methods may indeed be fruitful, as the process of manual tuning of GHMM parameters (i.e., "tweaking") after MLE training is commonly acknowledged by those with experience training GHMM-based gene finders (including our own systems). The practice of performing such tuning on the training set, especially when done iteratively, can be viewed as a manual form of gradient ascent optimization using the percentages of correctly predicted nucleotides, exons, and whole genes as surrogates for the Σ_(S,φ)∈T _P(φ|S,θ) term in Equation 2.

We therefore decided to investigate the use of a simple form of global discriminative training for gene-finding. We did this by building a rudimentary gradient ascent optimizer and applying it to a subset of the model parameters for our GHMM-based gene finder, TigrScan, as described in the Methods.

## Results

### Maximum likelihood versus discriminative training

Results for *Arabidopsis thaliana *are shown in Table 1 and those for *Aspergillus fumigatus *are shown in Table 2. The two methods being compared are maximum likelihood estimation (MLE) versus maximum likelihood followed by gradient ascent parameter estimation (GRAPE).

**Table 1:** Results on *Arabidopsis thaliana*

method	train	test	nucAcc	exonF	geneSn
GRAPE	CV	CV	95 ± 1%	82 ± 2%	49 ± 3%
GRAPE	CV	H	93 ± 1%	80 ± 2%	44 ± 3%
GRAPE	T	T	95%	86%	57%
GRAPE	T	H	94%	81%	48%
MLE	CV	CV	90 ± 1%	72 ± 2%	33 ± 4%
MLE	T	T	91%	75%	36%
MLE	T	H	90%	71%	33%

GRAPE = GRadient Ascent Parameter Estimation, MLE = Maximum Likelihood Estimation only. CV=cross validation, T = training set, H = 1000-gene hold-out ("test") set. CV in the *train *column means training on 800 genes from T. CV in *test *column means testing on 200 genes from T. In rows with a CV in either column, numbers are averages from 5 runs. nucAcc = nucleotide accuracy, exonF = exon F score, geneSn = gene sensitivity. F = 2SnSp/(Sn+Sp) for Sn = sensitivity and Sp = specificity. CV averages are reported ± SD.

**Table 2:** Results on *Aspergillus fumigatus*

method	train	test	nucAcc	exonF	geneSn
GRAPE	CV	CV	88 ± 1%	54 ± 4%	35 ± 4%
GRAPE	CV	H	88 ± 1%	51 ± 2%	29 ± 1%
GRAPE	T	T	92%	65%	48%
GRAPE	T	H	87%	51%	31%
MLE	CV	CV	81 ± 3%	27 ± 8%	16 ± 5%
MLE	T	T	88%	42%	28%
MLE	T	H	83%	30%	18%

See Table 1 for legend.

The *train *column indicates whether training (i.e., parameter estimation) was performed on the entire training set (T) or on separate 800-gene cross-validation partitions (CV). The *test *column indicates whether accuracy was measured on the full training set (T), on one-fifth of the training set (CV), or on the unseen data (H). We will consider the evaluation on H to be the most reliable measure of gene finder accuracy. For any row containing a CV, we report the average of five runs, where each run used a different 800-gene subset of the training data for parameter estimation.

**Figure 1:**
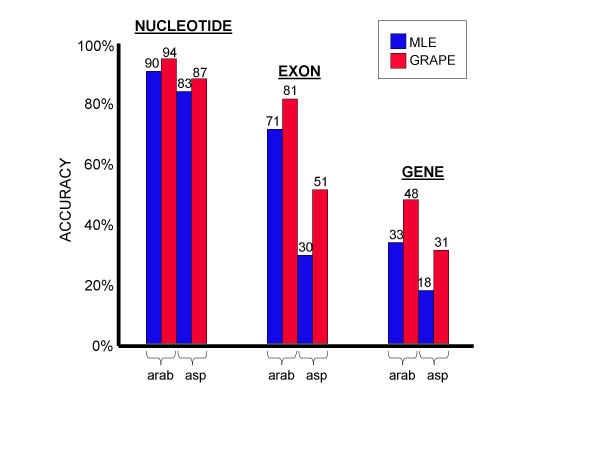
**Maximum likelihood versus gradient ascent **Gradient ascent parameter estimation (GRAPE) improves accuracy over MLE at the nucleotide, exon, and whole gene levels. arab = *Arabidopsis thaliana*, asp = *Aspergillus fumigatus*.

Both tables give compelling evidence for the value of gradient ascent training, as shown in Figure 1. In *Arabidopsis*, gradient ascent applied to the full training set improved over the MLE method from 71% to 81% at the level of exons and 33% to 48% at the level of whole genes. In *Aspergillus *the improvement was even more dramatic: 30% to 51% at the exon level and 18% to 31% for whole genes. A gain of 4% nucleotide accuracy was measured for both organisms.

### Data partitioning and cross validation

**Figure 2:**
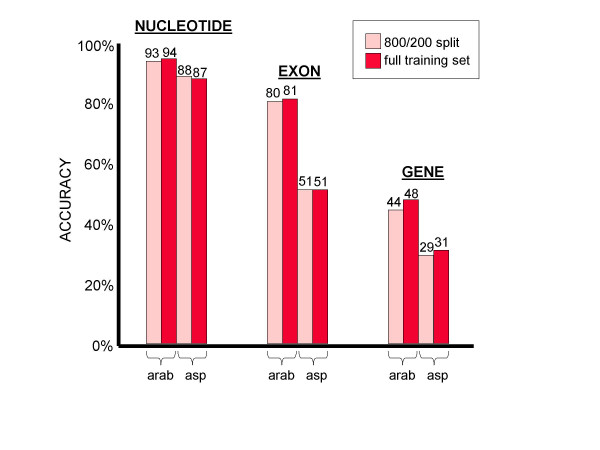
**Data partitioning for gradient ascent **Separating the training set into an 800-gene MLE set and a 200-gene gradient ascent set provides no improvement over simply performing MLE and GRAPE on the full training set.

A tangible improvement was still seen when a cross-validation design was used to split the training set so as to separate the data used for maximum likelihood estimation (800 genes) and subsequent gradient ascent (200 genes). However, results from both organisms suggest that this separation did not improve the accuracy of the gene finder, as shown in Figure 2. Indeed, on *Arabidopsis*, gradient ascent training produced greater gains in accuracy when performed on the entire training set rather than using the cross-validation structure, while on *Aspergillus *the improvement due to using a cross-validation structure was either small (nucleotide level: 1%), zero (exon level), or negative (gene level: -2%). Thus, the recommended training protocol would be to apply MLE to the entire training set followed by gradient ascent on the full training set as well.

**Figure 3:**
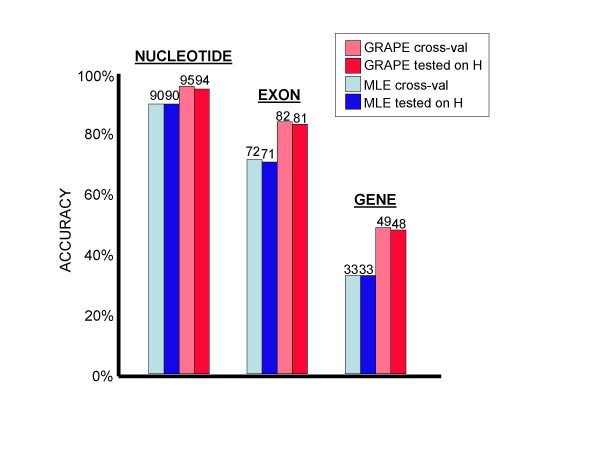
**Cross-validation versus testing on unseen data **Cross-validation scores provide a reasonably accurate prediction of performance on unseen data. Results shown for *A. thaliana *only; results for *A. fumigatus *are given in Table 2.

Although use of a cross-validation structure to split the training set for the twin purposes of maximum likelihood estimation of ~90,000 parameters and gradient ascent refinement of 29 parameters is therefore not justified (according to the above results), cross-validation does seem to have some value in terms of predicting how well the gene finder will perform on unseen data, as suggested by Figure 3.

On both genomes and at all levels (nucleotide, exon, gene), accuracy measurements obtained through cross-validation were closer to the accuracy measured on unseen data than were the measurements taken from the full training set, as we expected. This was true both with and without gradient ascent, though when gradient ascent was applied, even the cross-validation results were slightly inflated. The latter observation is presumably attributable to the "peeking" that was permitted (see Methods), whereby the gradient ascent procedure received feedback from the 200 evaluation genes held out from the training set, T. This suggests that estimating even small numbers of parameters (in this case 29) from the test set can artificially inflate accuracy measurements on that set.

**Figure 4:**
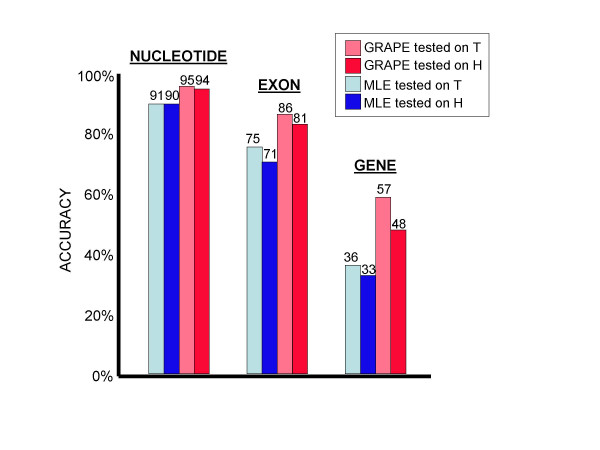
**Evaluation on the training set **Accuracy measurements taken from the training set were artificially inflated, as expected. Results are shown only for *A. thaliana*; results for *A. fumigatus *were even more extreme.

Figure 4 illustrates the effects of testing the gene finder on the training set. As can be seen from the figure, the accuracy measurements taken from the training set can be substantially inflated relative to the more objective measurements taken from the hold-out set, thereby promoting overly optimistic expectations for how the gene finder will perform on unseen data.

## Discussion

The results presented above provide a clear demonstration that independent maximum likelihood estimation of submodel parameters is sufficiently neglectful of global GHMM behavior as to compromise gene finder accuracy. Even such a crude method as our 29-parameter gradient ascent procedure proved to be effective at significantly improving accuracy over that achievable by simple MLE training. The potential for more sophisticated global discriminative training methods to produce even greater improvements is surely worthy of investigation.

It is interesting to observe that the natural language processing and speech recognition communities, from whom HMM-based methods were originally borrowed for use in bioinformatics, have been moving toward global discriminative training methods for some time. The two most popular forms of discriminative training for speech recognition are Maximum Mutual Information (MMI) and Minimum Classification Error (MCE). Both methods can be implemented using an iterative gradient ascent/descent algorithm. Our approach is most similar in spirit to that of MCE.

In the case of "pure" (i.e., non-generalized) HMMs, expectation-maximization (EM) update formulas have been derived for both MMI and MCE. These formulas allow model parameters to be updated in an *axis-oblique *(rather than *axis-parallel*) manner; i.e., multiple parameters can be adjusted simultaneously, so that the optimizer is less constrained in following the direction of steepest gradient in parameter space. This may reduce the number of steps required for convergence. Indeed, more rapid convergence (in terms of numbers of re-evaluation steps) has been cited as a concrete advantage of these EM-style formulations over more generalized gradient ascent methods [23]. However, EM-style approaches to the discriminative training problem for HMMs have typically involved a number of simplifying assumptions and/or heuristics, thereby voiding formal assurances of optimality (e.g., [17,24,18,26]). Furthermore, as with more generalized gradient ascent procedures, EM often tends to find only a local optimum rather than a global one [13].

In the case of GHMM-based gene finders, the advantages of EM over a generalized gradient ascent procedure may indeed be rather slim. The very flexibility which we find attractive in GHMMs can be expected to complicate the derivation of such EM-like update formulas for arbitrary GHMM-based gene finders, likely requiring additional assumptions and approximations that would further compromise the optimality of the EM procedure. It was for this reason that we decided to employ a more generalized gradient ascent method for the present study. A rudimentary gradient ascent optimizer is simple to implement, and the use of prediction accuracy as an objective function affords great convenience in approximating Σ_(S,φ)∈T_P(φ|S,θ). Although P(φ|S,θ) can be more directly computed using a modified Forward algorithm [23], to do so would in theory be no more efficient than running the full gene finder, since the asymptotic run times of the Forward and Viterbi algorithms for GHMMs are equivalent. Nevertheless, inasmuch as the Forward algorithm provides a more direct approximation of P(φ|S,θ), its use for this purpose is worthy of investigation.

There are a number of other variations and enhancements which we are at present contemplating for our discriminative trainer. One of these involves the joint training of pairs of submodels in the GHMM using a maximum discrimination criterion rather than the usual one based on maximum likelihood. Although such an approach would not in itself directly attend to the global optimality of the GHMM (indeed, we already apply such an approach to our signal sensors during our so-called "MLE" training regime, as remarked earlier), it would at least seem to offer a promising direction for improving our existing optimizer and may be feasible without increasing the computational cost beyond what is practical.

For the present, we feel confident in making the recommendation that others tasked with the training of GHMM gene finders consider applying an automated gradient ascent procedure like that described here as a more systematic alternative to manual tuning of parameters following maximum likelihood training of individual submodels. Beyond the obvious advantage of likely improving gene finder accuracy, such an automated method may offer some degree of reproducibility (notwithstanding the typically stochastic nature of such methods) and uniformity for the purposes of comparing gene finders and gene finding algorithms. In addition, we urge those practicing manual tuning on their final "test" set to consider that their reported accuracy results may well be inflated as a result of "peeking" at the test set before the final evaluation – a practice that has been criticized in the field of machine learning (eg., [27]). That significant inflation was seen in our studies as a result of tuning only 29 of the ~90,000 GHMM parameters on the 200-gene "test" set suggests that the phenomenon may conceivably occur to some degree even when an automated procedure is not employed.

Finally, we would like to make note of an unfortunate consequence of discriminative training of HMMs for biological sequence analysis, namely, that while the resulting models may possess improved ability for discrimination and therefore greater utility for specific tasks such as gene prediction, their suitability as representative models of biological knowledge (especially probabilistic knowledge) may well be reduced relative to models induced with simple MLE techniques. Indeed, some authors in the field of speech recognition (e.g., [20]) have noted that more accurate discrimination can sometimes be obtained by relaxing sum-to-one constraints for probability distributions, thereby permitting the gradient ascent procedure to automatically discover appropriate weightings between states or inputs. This is reminiscent of the exon "optimism" parameter which we employ and which seems to have no principled justification (and indeed, we might speculate that this extraneous parameter proved useful precisely because it enabled a primitive form of discriminative training by providing an explicit "correction factor" or weighting between submodels). Thus, despite the apparent value of discriminative training in improving gene finder accuracy, our ability to extract biological knowledge by inspecting the parameters of a gene finder trained in this way may be somewhat hindered. For the present, this does not seem to be of great practical significance, but it is a consideration worthy at least of mention.

## Conclusions

We have shown that discriminative training for GHMM-based gene finders is feasible using a rudimentary gradient ascent approach, and have briefly explored the relation between this method and the EM-like techniques which have been proposed in the field of speech recognition. Our experiments show that the gradient ascent method can result in a gene finder with substantially greater prediction accuracy. It is our hope that even greater gains in accuracy will result from extension and refinement of discriminative training techniques applied to GHMM-based gene finders.

## Methods

### Description of the GHMM

The gene finder TigrScan [8] is a GHMM-based program similar to Genie [1] and Genscan [2,28]. The forward-strand model contains six signal states (donor and acceptor sites; start and stop codons; promoter; poly-A signal) and eight content states (intron; intergenic; 5' and 3' UTR; initial, internal, final, and single exons). The reverse-strand model mirrors that of the forward strand. Four relative frequency histograms are used to estimate the duration probabilities of the four exon types; the four noncoding states are assumed to have geometric duration distributions and are therefore each parameterized by a single value representing the mean duration. Each content state is scored using a separate fifth-order Interpolated Markov Model (IMM) [29]. TigrScan offers a number of signal sensors, including WMMs, WAMs, WWAMs, and MDD trees [28] having any of the foregoing signal sensors as leaf models; for this study we used only (non-MDD) WAMs, though the order of the Markov chains within the WAMs was allowed to vary. Putative signals scoring below a given signal threshold are ignored by TigrScan. This threshold is chosen separately for each signal sensor so as to achieve a desired sensitivity *Sn *(*Sn *= *TP*/(*TP*+*FN*), *TP *= true positive count, *FN *= false negative count) on a training set of true and "decoy" signals. "Boosting" of signal sensors was performed by iteratively retraining each signal sensor on sets of training features in which the lowest scoring features were duplicated so as to focus the training procedure on the most difficult examples. Boosting has been found to improve signal detection in other application areas [30]. Most transitions in the GHMM are obligatory (such as "donor site → acceptor site"); of the non-obligatory transitions, sum-to-one constraints and the forward/reverse strand equivalence reduce the number which can be independently varied to just four. Transitions into exon states are modified by an exon "optimism" multiplier (similar to that described in [6]) which has been seen anecdotally to be useful in improving prediction accuracy (unpublished data).

### Parameters to be optimized

The total number of parameters which need to be estimated when training TigrScan is roughly 90,000; the large bulk of these are the n-gram statistics comprising the IMMs used for the content sensors. As an initial attempt at applying discriminative training to TigrScan, we selected 29 of these ~90,000 parameters to subject to gradient ascent optimization. Although this is a miniscule proportion of the available parameters, our previous experiences with hand-tuning our GHMM on other data sets suggested that these 29 parameters exert a disproportionately large influence on the accuracy of the gene predictions. By limiting the number of parameters to be optimized we hoped to both accelerate the training procedure and also reduce the risk of overtraining. The selected parameters were:

• mean intron, intergenic, and UTR lengths (3)

• transition probabilities (4)

• exon optimism (1)

• WAM size and relative positioning (8)

• WAM order (4)

• signal sensitivity (1)

• number of signal boosting iterations (8)

• skew and kurtosis of exon length distributions

Modifications to skew and kurtosis of exon length distributions were found during early exploration to produce no improvements; these parameters were therefore left unchanged in all further experiments. All remaining parameters were estimated using standard MLE techniques.

For those runs in which gradient ascent was disabled (see below), the following methods were used to estimate the above 29 parameters: mean intron and UTR lengths as well as transition probabilities were estimated using MLE from training data; mean intergenic length was set to a fixed value based on the known intergenic lengths in the test set; exon optimism was set to zero; remaining parameters were selected so as to minimize the misclassification rate on a set of true and "decoy" signals selected from the training set.

### Objective function and optimization procedure

As an *objective function *for use by the gradient ascent procedure, we decided to measure the accuracy of the current parameterization by running the gene finder on a subset of the training genes. Our hope was that this accuracy measure would provide a reasonable approximation of Σ_(S,φ)∈T _P(φ|S,θ) by indicating roughly how often the current model θ would cause the correct parse φ to be predicted for training sequence S. We defined the nucleotide accuracy *A*_*nuc *_as the percentage of nucleotides correctly classified as coding vs. noncoding; *A*_*exon *_was defined as an average of exon sensitivity and specificity (where a predicted exon is considered correct only if both boundary coordinates were predicted correctly); and *A*_*gene *_was defined as the percentage of training genes which were predicted exactly correctly. These were all rounded to integral percentages between 0 and 100%. The objective function was then defined as:

*f*(θ) = 100*A*_*nuc*_+*A*_*exon*_+*A*_*gene*_.    (4)

The *A*_*nuc *_and *A*_*exon *_terms were included in an effort to smooth the function, which would otherwise have been insensitive to changes not reflected in the number of genes predicted exactly correctly – i.e., a step function. Though the *A*_*nuc *_term was given much greater weight for this study, additional work needs to be undertaken to determine the most suitable set of weights for our objective function.

**Figure 5:**
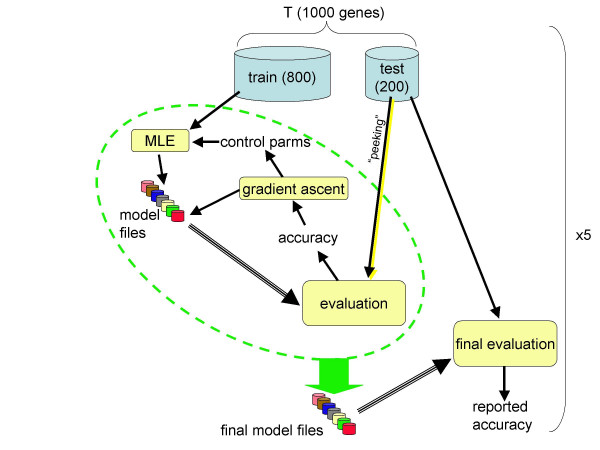
**Gradient ascent training **Schematic diagram of gradient ascent training procedure. Of 29 parameters modified by gradient ascent, some (e.g., WAM size) were used to control the MLE estimation procedure, while others (e.g., mean intron length) were used directly as parameters to the GHMM. Testing of the gradient direction was performed on the 200-gene cross-validation set, which was part of the 1000-gene training set, T.

Parameters were optimized using an iterative gradient ascent procedure operating in the selected 29-dimensional parameter space, as illustrated schematically in Figure 5. Steps were taken in an axis-parallel manner (one step per axis per iteration), with the step size for each axis decreasing by half whenever a local maximum was reached on that axis.

### Data and experimental design

The quality of a given parameterization θ was measured by evaluating the objective function *f*(θ) on a held-out subset of the training set. The training set was limited to 1000 genes, and all experiments were repeated separately on two highly divergent species, the model plant *Arabidopsis thaliana *and the pathogenic fungus *Aspergillus fumigatus*. Five-fold cross-validation was employed, so that the entire optimization procedure was carried out five times on four-fifths of the data (800 genes) and each time evaluated on the remaining one-fifth (200 genes); accuracy results reported here were obtained by averaging the five sets of accuracy numbers obtained from the cross-validation.

**Figure 6:**
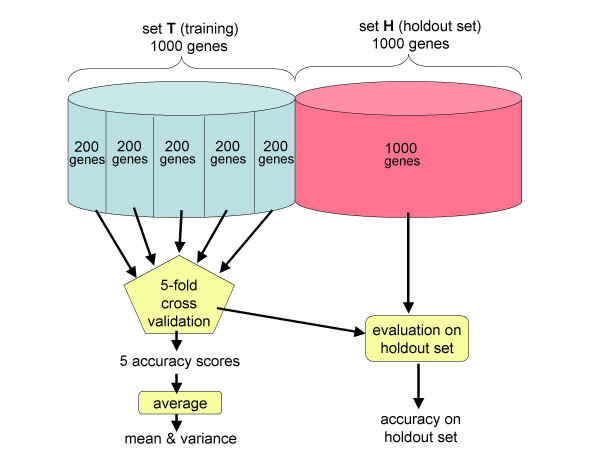
**Cross-validation experiments **Five-fold cross-validation was used both in the gradient ascent and in the MLE-only experiments. For gradient ascent training, MLE was performed on four-fifths of the training set (T) and then gradient ascent was performed on the other one-fifth. A separate hold-out set (H) of 1000 genes was used to obtain an unbiased evaluation of all final models.

The held-out one-fifth was also used by the gradient ascent procedure to tune the selected 29 parameters. The practice of using a held-out set for smoothing or to estimate a small number of additional parameters is common in the natural language processing field [31], where it is recognized that such "peeking" at the test set (by which we mean iterative re-estimation of model parameters from the training set after receiving accuracy feedback on the test set) by the training procedure can (unfortunately) artificially inflate reported accuracy numbers. For this reason, an additional 1000 genes were used for testing the gene finder after each cross-validation run. The results of this final testing were *not *made available to the optimizer, but are instead reported here as a more objective assessment of final model accuracy. We will refer to the training set as T and the additional 1000 genes for testing as H. BLAST [32] was used to ensure that no two genes in T∪H were more than 80% similar over 80% of their lengths at the nucleotide level. This training protocol is illustrated in Figure 6.

Several variations of this experiment were also performed. To evaluate the utility of splitting the training set and performing MLE and gradient ascent parameter estimation on separate subsets (as described above), we also performed MLE followed by gradient ascent training on the full training set T and again evaluated the induced models on H. To assess whether gradient ascent provided any improvement in accuracy we also trained a model on T using only MLE and evaluated that model on H. Although the virtues of cross-validation have been well explored in the context of many other applications, we decided to use the above experimental design as a convenient opportunity to verify our expectation that it would also prove useful for objective analysis of gene finder accuracy.

## Authors' contributions

Software implementation and computational experiments were performed by WHM. The manuscript was written by WHM with assistance from SLS.
