# An empirical analysis of training protocols for probabilistic gene finders

**DOI:** 10.1186/1471-2105-6-193

**Published:** 2005-08-01

**Authors:** William H Majoros, Steven L Salzberg

**Affiliations:** 1 The Institute for Genomic Research, 9712 Medical Center Drive, Rockville, MD 20850, USA

The summands in Equations (1)–(3) in the original paper [1] should be logarithmic. The corrected equations are given below.







Any references to these equations appearing in the text should be modified accordingly.
